# Puquitinib mesylate, an inhibitor of phosphatidylinositol 3-kinase p110δ, for treating relapsed or refractory non-Hodgkin's lymphoma

**DOI:** 10.18632/oncotarget.5833

**Published:** 2015-10-17

**Authors:** Hang Yang, Yu Wang, Jing Zhan, Yi Xia, Peng Sun, Xi-Wen Bi, Pan-Pan Liu, Zhi-Ming Li, Su Li, Ben-Yan Zou, Wen-Qi Jiang

**Affiliations:** ^1^ Sun Yat-sen University Cancer Center, State Key Laboratory of Oncology in South China, Collaborative Innovation Center for Cancer Medicine, Guangzhou 510060, China; ^2^ Department of Medical Oncology, Sun Yat-sen University Cancer Center, Guangzhou 510060, China; ^3^ Clinical Trial Center, Sun Yat-sen University Cancer Center, Guangzhou 510060, China; ^4^ Nursing Department, Sun Yat-sen University Cancer Center, Guangzhou 510060, China

**Keywords:** XC-302, PI3K pathway, non-hodgkin lymphoma, safety and efficacy, pharmacokinetics

## Abstract

**Objectives:**

To determine the safety of Puquitinib Mesylate (XC-302), an oral inhibitor of phosphatidylinositol 3-kinase, in treating relapsed or refractory non-Hodgkin's lymphoma (NHL).

**Methods:**

Between October 2013 and July 2015, 21 patients from Sun Yat-sen University Cancer Center were treated twice daily on each day of a 28-day cycle (median number of cycles, 2; maximum, 20) with XC-302 at a post prandial dose of 25 mg, 37.5 mg, or 50 mg. Adverse events (AEs), AUC_last_ and C_max_, response rates, and overall survival were assessed.

**Results:**

Patients had received a median (range) of 1 (1 to 3) previous cancer treatments. At the latest follow-up, two patients were still benefitting from the study. The most common drug-related AEs were elevations in alanine transaminase (ALT, 14 of 21 patients) and aspartate transaminase (AST, 7 of 21 patients). Four patients, both in the-50-mg group, had dose-limiting toxicities, and therapy was discontinued in a fifth because of persistent abnormal liver function. The overall response rate was 2 of19. Serum concentrations of XC-302 increased in a dose-dependent pattern. Median progression-free survival in all patients was 1.9 (95% CI, 1.7 to 2.0) months.

**Conclusion:**

XC-302 has an acceptable safety profile and offers potential therapeutic value to patients with relapsed or refractory non-Hodgkin lymphoma.

## INTRODUCTION

The phosphatidylinositol 3-kinase (PI3K) signaling pathway is an essential component of malignant cellular processes [1–3] that is critical to the development and progression malignant tumors [4]. The inhibitors targeting PI3K-pathway proteins have been investigated as new anti-cancer drugs [5–7], and several are currently in early phase clinical trials [6, 8].

Puquitinib Mesylate (XC-302), developed independently by Xinchang Pharmacy Corporation (Zhejiang Medicine Co., Ltd. China), is a new multiple-target-point inhibitor. Preliminary studies have discovered that XC-302 inhibits the proliferation of several cancer cell lines, such as colon, lung, breast, ovarian, prostate cancer, lymphoma, leukemia, osteosarcoma, in which the half maximal inhibitory concentration (IC_50_) ranged between 0.5 and 2.0 μM.

Cell lines with drug resistance mediated by p170/MDR-1 / p-Glycoprotein are similarly sensitive to XC-302. It also exhibited obvious anti-tumor efficacy in xenografts to nude mice with cancer of the colon, stomach, or lung. XC-302 immediately suppresses the activity of PI3K (in subtype IA, IC_50_ against PI3K isoforms was p110α = 766.6 nM, p110β = 699.4 nM, p110δ = 2.8nM, and p110γ = 89.7 nM). It also apparently inhibits AKT phosphorylation mediated by EGFR (IC50 = 0.1 μM), the activity of receptor tyrosine kinase (KDR and PDGFR β; IC_50_ = 1.0 μM), and the formation of the vascular endothelial cells lumen (IC_50_ = 0.1 μM). In addition, it is rapidly absorbed and has a high absolute bioavailability, as indicated by the pre-clinical pharmacokinetics tests. Finally, XC-302 shows low toxicity by toxicology experiments (Data were quoted from the unpublished information, personal communication with Wei Mao on January 1, 2013).

In a first-in-man Phase I study in Chinese patients with advanced solid tumors, the dosing was set on the basis of efficacy in animal models and toxicity studies. The maximum tolerated dose (MTD) of single-agent XC-302 was 75 mg when given on a continuous daily schedule postprandial and 50 mg when given twice daily (BID) postprandial. Dose-limiting toxicities (DLT) included reversible increased ALT/AST concentrations and vomiting. XC-302 exhibited anti-cancer activity in advanced solid tumors (disease control rate = 52.5%). Clinical pharmacokinetic (PK) monitoring revealed good oral bioavailability (Data were quoted from the unpublished information, personal communication with Wei Mao on January 1, 2013).

On the basis of these data, we conducted a phase 1, dose-escalation study of XC-302 in patients with relapsed non-Hodgkin's lymphoma (NHL). Our objectives were to determine the safety, pharmacokinetics characteristics, and efficacy of the drug.

## RESULTs

The first patient was enrolled in October 2013, and the last follow-up visit was completed in July 2015. Follow-up ranged from 0.2 to 19.3 months, with a median of 1.7 months (SD = 4.4); two patients (9.5%) were lost to follow-up. Median (range) age of the 21 patients (17 men) was 56 years (39 to 72 years). All patients had Eastern Cooperative Oncology Group performance scores of 1. All had received standard treatment(s) before the study with little effect.

Of the 21, 3 received 25 mg doses of XC-302, 9 received 37.5 mg, and 9 received 50 mg. The median number of cycles received was 2 (range 1 to 20). The most common primary tumors were small lymphocytic lymphoma/chronic lymphocytic leukemia (SLL/CLL), which were found in 10 patients and mantle cell lymphoma, which was found in 5 (Table 1).

**Table 1:** Baseline Characteristics of Patients with Relapsed or Refractory Non-Hodgkin's Lymphoma in a Phase I Trial of Puquitinib Mesylate (XC-302, an inhibitor of phosphatidylinositol 3-kinase p110δ)

Characteristic	XC-302 dosage			
	25 mg, BID *n* = 3	37.5 mg, BID *n* = 9	50 mg, BID *n* = 9	All *n* = 21
**Age, median (range), y**	54 (43–58)	49 (39–72)	58 (42–68)	56 (39–72)
**Sex, *n* (%)**				
Men	2 (66.7)	7 (77.8)	8 (88.9)	17 (81.0)
Women	1 (33.3)	2 (22.2)	1 (11.1)	4 (33.3)
**ECOG performance status, n (%)**				
0	0	0	0	0
1	3 (100)	9 (100)	9 (100)	21 (100)
**Disease type, *n* (%)**				
Follicular lymphoma	1 (33.3)	1 (11.1)	1 (11.1)	3 (14.3)
SLL/CLL	0	1 (11.1)	5 (55.6)	10 (47.6)
Mantle cell lymphoma	2 (66.7)	5 (55.6)	2 (22.2)	5 (23.8)
Peripheral T-cell lymphoma	0	1 (11.1)	0	1 (4.8)
BPDCN	0	1 (11.1)	0	1 (4.8)
Gastric MALT	0	0	1 (11.1)	1 (4.8)
**Bulky disease (≥1 lymph node ≥5 cm), *n* (%)**	1 (33.3)	4 (44.4)	2 (22.2)	7 (33.3)
**Elevated lactate dehydrogenase, *n* (%)**	0	4 (44.4)	3 (33.3)	7 (33.3)
**Anemia, *n* (%)**	0	1 (11.1)	3 (33.3)	4 (33.3)
**Thrombocytopenia, *n* (%)**	1 (33.3)	5 (55.6)	3 (33.3)	9 (42.9)
**Neutropenia, *n* (%)**	1 (33.3)	2 (22.2)	3 (33.3)	6 (28.6)
**Elevated ALT/AST, *n* (%)**	1 (33.3)	2 (22.2)	0	3 (14.3)
**Clinical stage, *n* (%)**				
III	0	1 (11.1)	4 (44.4)	5 (23.8)
IV	3 (100)	8 (88.9)	5 (55.6)	16 (76.2)
**LBMI, *n* (%)**	3 (100)	6 (66.7)	4 (44.4)	13 (61.9)
**Time since diagnosis, median (range), months**	38.1(1.3–63.3)	18.4(4.4–113.9)	36.0(3.9–71.9)	29.7(1.3–113.9)
**Prior therapies, median (range)**	1 (1–3)	1 (1–3)	1 (1–3)	1 (1–3)
**Prior therapy type, *n* (%)**				
Rituximab	2 (66.7)	4 (44.4)	8 (88.9)	14 (66.7)
Alkylating agent	3 (100)	9 (100.0)	9 (100.0)	21 (100)
Anthracycline	3 (100)	7 (77.8)	6 (66.7)	16 (76.2)
Bortezomib	1 (33.3)	0	0	1 (4.8)
Purine analog	1 (33.3)	4 (44.4)	6 (66.7)	11 (52.4)

ECOG, Eastern Cooperative Oncology Group; SLL/CLL, small lymphocytic lymphoma/chronic lymphocytic leukemia; BPDCN, blasticplasmacytoid dendritic cell neoplasm; LBMI, lymphoma bone marrow involvement; ALT/AST, alanine transaminase (ALT) and aspartate transaminase

### Safety and toxicity

All patients experienced at least one drug-related AE during the trial (Table 2). Most adverse events were grade 1 or 2 and resolved with appropriate management. The most common AEs were elevated alanine transaminase (ALT, 66.7%) and aspartate transaminase (AST, 33.3%) concentrations, anemia (33.3%), neutropenia (23.8%), leukopenia (19.9%) and thrombocytopenia (19.0%). However, the marrow suppression was considered not correlated with XC-302 after the judgment. Other familiar AEs included upper respiratory infection (URI, 19.0%), gastrointestinal adverse reactions, fecal occult blood (19.0%), hematuresis (14.3%), creatinine elevation (9.5%), myocardial ischemia (9.5%), peripheral nerve toxicity, rash (9.5%) and so on which were responded well to symptomatic treatment and were generally well tolerated.

**Table 2:** Frequency of Adverse Events Experienced by More Than One Patient

XC-302 dose, BID							Overall	
Event	25 mg, *n* = 3		37.5 mg, *n* = 9		50 mg, *n* = 9			
	Any, *n*	≥3, *n*	Any, *n*	≥3, *n*	Any, *n*	≥3, *n*	Any, *n*	≥3, *n*
Nausea	…	…	1	…	1	…	2	…
Diarrhea	…	…	…	…	2	…	2	…
Constipation	…	…	1	…	1	…	2	…
Fecal occult blood	1	…	2	…	1	…	4	…
Pyrexia	…	…	2	…	2	…	4	…
URI	…	…	2	…	2	…	4	…
Rash	…	…	…	…	2	…	2	…
Creatinine, increased	1	…	…	…	1	…	2	…
Proteinuria	…	…	1	…	…	…	1	…
Hematuresis	1	…	1	…	1	…	3	…
AST, increased	…	…	1	…	6	1	7	1
ALT, increased	2	…	5	…	7	4	14	4
S-T-segment change	…	…	1	…	1	…	2	…
Arrhythmia	…	…	…	…	1	…	1	…
Peripheral nerve toxicity	…	…	…	…	1	…	1	…
Anemia	1	…	4	1	2	…	7	…
Thrombocytopenia	1	…	2	1	1	1	4	2
Neutropenia	…	…	2	…	3	2	5	2
Leukopenia	1	…	1	…	2	…	4	…

Four patients (No. 9, 14, 15, 21), all receiving 50 mg, had a grade 3 transaminases elevation, which was considered to be related to XC-302, and one of these (No. 15) was withdrawn when the elevation continued (Table 2). These AEs met the definition of a DLT. Therefore 50 mg twice a day was the determined MTD for this schedule. No DLT events were observed at the 25-mg or 37.5-mg doses.

### Pharmacokinetics of XC-302

**Figure 1:**
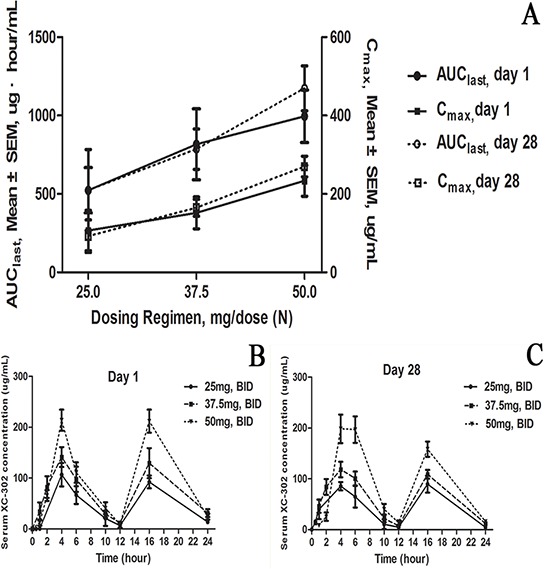
Dose-exposure and serum profile of XC-302 in 21 patients with relapsed or refractory non-Hodgkin's lymphoma **A.** Steady-state (day 1 and day 28) XC-302 plasma exposures by XC-302 dosing regimen (*N* = 21). AUC_last,_ area under the curve from time zero to the last detectable value; C_max_, maximum XC-302 concentration. Mean plasma concentration–time profiles, **B.** day 1 and **C.** day 28. SEM, standard error of the mean.

Pharmacokinetic evaluations were performed on all patients. Trough values for XC-302 were similar on days 1 and 28, demonstrating that a steady state was achieved. Pharmacokinetic response was linear, with general exposure to XC-302 (AUC_last_ and C_max_) increasing with increasing dose. Similar pharmacokinetic values were observed on day 28 (Figure 1). XC-302 serum concentrations peaked about 4 hours after administration and decreased slowly (Figure 1). Serum XC-302 concentrations generally increased in a dose-dependent manner over the investigated range (doses ranged from 25 mg to 50 mg).

### Antitumor activity

**Figure 2:**
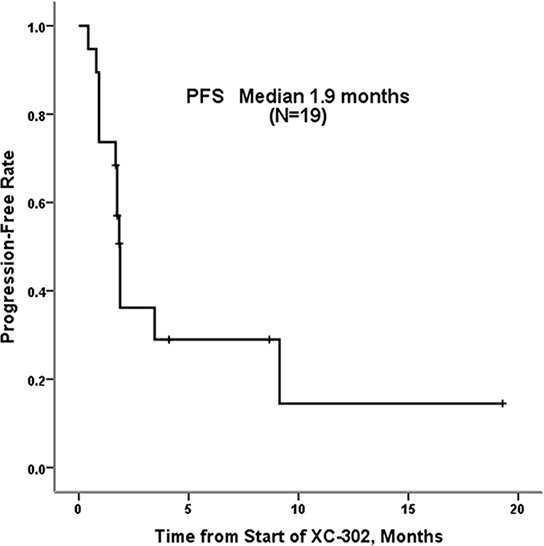
Progression-free survival for 21 patients with relapsed or refractory non-Hodgkin's lymphoma treated with XC-302 Median progression-free survival was 1.9 months.

At least one response assessment was available for 19 patients (Table 3). Two patients were loss to follow-up and withdrawn from the study. At the first efficacy evaluation, 6 patients had stable disease and 11 had progressive disease. The overall response rate was zero in patients taking the 25-mg or 37.5-mg doses (Table 3). Only 2 of 19 achieved an objective response, one partial response and one minor response. Time to response for these two patients was 1.8 and 1.84 months. For all 19 patients, median (range) duration of response was 7.1 (6.9 to 7.3) months, and the median (range) progression free survival (PFS) was 1.9 (95% CI, 1.7 to 2.0) months (Figure 2). The range of progression-free survival was 1.5 to 1.9 months for the 12 patients taking the 25-mg and 37.5-mg doses and 0.9 to 9.1 months for the 7 patients taking the 50-mg dose.

**Table 3:** Response to Treatment with XC-302 among 21 Patients with relapsed or Refractory Non-Hodgkin's Lymphoma

Response	XC-302 dose, BID			All (*n* = 21), *n*
	25 mg (*n* = 3), *n*	37.5 mg (*n* = 9), *n*	50 mg (*n* = 7*), *n*	
**Overall response rate**	0	0	2	2
**Complete response**	0	0	0	0
**Partial response**	0	0	1	1
**Minor response**	0	0	1	1
**Stable disease**	3	1	2	6
**Progressive disease**	0	8	3	11

* Two patients lost to follow-up during the study are not included in this analysis.

At the latest follow-up, two patients were still on treatment. One, with gastric mucosa-associated lymphoid tissue in the 50-mg group, achieved a partial response, and the other, with mantle cell lymphoma in 25 mg group, had stable disease. Among SLL/CLL patients receiving a dose of 50 mg, one had a partial response after the 4th treatment cycle but progressive disease after the 12th cycle.

## DISCUSSION

In this phase 1 study, XC-302 was well tolerated, and most treatment-emergent AEs were grade 1 or 2 in severity, suggesting that XC-302 will be compatible in combination with cytotoxic agents or other targeted agents. The most common AEs were abnormal liver function (increased ALT/AST concentrations) and myelosuppression. Others included adverse gastrointestinal reactions, hematuresis and rash, which have been reported in other study of PI3K inhibitors [9–11]. However, in the 13 patients (61.9%) with stage IV disease with lymphoma bone marrow involvement and hemopoietic malfunction, myelosuppression was judged not to be related to XC-302 therapy. In addition, many other AEs (eg, infection, nausea, constipation) were expected because intercurrent illness in an older population of patients, and side effects of previous therapy.

PI3K signaling disrupts insulin signaling, and hyperglycemia has been considered a toxicity of PI3K inhibition [8, 12]. However, we observed no XC302-related hyperglycemia. One possible explanation is that serum glucose concentrations are characteristically elevated in patients receiving pan-PI3K inhibitors that have activity against PI3Kα [8, 13], whereas XC-302 showed a selectivity for PI3Kδ. PI3Kδ is an important messenger in B-cell receptor (BCR) signaling, and PI3Kδ signaling pathways are commonly overactive in B-cell tumors [14, 15]and increase proliferation, development and survival of tumor cells [16, 17]. Another possible explanation is that hyperglycemia was transient and did not markedly disturb glucose homoeostasis. The few patients with rashes did not require permanent drug discontinuation.

Treatment-specific grade 3/4 abnormal liver function without increased bilirubin concentrations was a DLT in four patients in the 50-mg group, and one was withdrawn because of refractory abnormal liver function. Liver toxicity is a class of adverse effects of PI3K/Akt/mTOR pathway inhibitors [9, 18–20]. Idelalisib is a potent and selective inhibitor of p110δ (IC_50_ = 8 nM) [15], and two phase I clinical trials reported asymptomatic grade 3 or greater serum transaminase elevations in 25% (*n* = 64) indolent non-Hodgkin lymphoma (iNHL) [21] and in 35% (*n* = 40) MCL [22] patients. Grade 3 AST abnormalities were observed in 2 of 84 patients with advanced solid tumors in a Phase I study of the PI3K inhibitor PX866 [9]. In another Phase I study of BGT226, in patients with advanced solid tumors and lymphoma, AST elevations were the most common biochemical abnormality (Grade 2, *n* = 9; Grade 3, *n* = 3) [18]. In clinical trials of BKM120, liver enzyme concentrations (including ALT/AST, transaminase, and hyperbilirubinemia levels) have been elevated with varying frequencies [20, 23].

The small sample size and the heterogeneity of the patients preclude any conclusions about the clinical activity of XC-302. The overall objective response we observed was lower than that of other PI3K p110δ inhibitors, such as Idelalisib [21, 22], perhaps because of insufficient cases, different types of disease, or the effectiveness of XC-302 itself. However, one of the nine patients with gastric mucosa-associated lymphoid tissue in 50-mg group achieved a PR in the first cycle. In the phase I study of Idelalisib in indolent non-Hodgkin lymphoma [21], the response rate in marginal-zone lymphoma subtype was 33%. The data in our research indicates that XC-302 might have the potential to be a promising therapy in patients with gastric MALT lymphoma.

## MATERIALS AND METHODS

### Ethics statement

We conducted this study in accordance with the guiding principles of the Declaration of Helsinki and after approved by the independent ethics committee of the Sun Yat-sen University Cancer Center. The study was registered with China Food and Drug administration (http://www.sda.gov.cn/WS01/CL0001/), certificate number 2007L04204. Written informed consent was obtained from each patient before enrollment.

### Patient population

Patients were eligible for the study if they were at least 18 years old and had a histologically confirmed diagnosis of lymphoma, including histologic subtypes of non-Hodgkin's lymphoma (NHL), treated at Sun Yat-sen University Cancer Center between October 2013 and July 2015. Patients had to be refractory to treatment or to have relapsed after standard therapy and had to have at least one lesion measuring 1.5 cm in a single dimension as measured on computed tomography (CT) or magnetic resonance imaging (MRI) scan. Patients had to have an Eastern Cooperative Oncology Group (ECOG) performance score no greater than 2 and be able to swallow tablets. All patients had to have adequate renal, liver function and appropriate hematologic values (WBC >1 × 10^9^/L, PLT > 50 × 10^9^/L) with a life expectancy of at least 3 months.

Patients were ineligible if they had symptomatic central nervous system metastasis or had received chemotherapy, radiotherapy, targeted therapy products (in the previous 4 weeks), PI3K inhibitors, strong CYP1A2 inhibitors, or strong inhibitors or inducers of CYP3A (a preliminary study showed that XC-302 irreversibly inhibited the activity of CYP1A2 enzyme). Patients testing positive for hepatitis B, hepatitis C (antibody), or human immunodeficiency virus (antigen or antibody), and those with serious other diseases, previous cancers, and gastrointestinal disease affecting oral absorption of drug were excluded. Pregnant or nursing women were also excluded.

### Study design and drug administration

This phase I study was designed as a single-arm, open-label dose-escalation study of XC-302. The MTD of XC-302 given daily as a single-agent after meals was 75 mg. A DLT greater than Grade 2 was not observed in patients treated with twice-daily dosing at 25 mg, 37.5 mg after the meal (28-day cycles). The data of PK and pharmacodynamics (PD) preclinical showed that dosing at 25 mg, 37.5 mg postprandial could suppress the concentration of lymphoma cell effectively (Data were quoted from the unpublished information, personal communication with Wei Mao on January 1, 2013).

At least three patients were enrolled in each dosage group. Starting at 25 mg, the dose was increased to 37.5 mg and then to 50 mg. XC-302 was administered orally BID on days 1–28 of a 28-day cycle until unacceptable toxicity or disease progression was observed. Escalation to the next highest dose was based on DLT observed at the previous dose.

### Research objectives

This was a phase I open label, dose-escalation study whose primary objective was to characterize the safety of XC-302 in adult Chinese patients with advanced lymphoma administered on a 28-day cycle. Others were to determine the pharmacokinetics (PK) characteristics and anti-tumor activity. The anti-tumor effects of XC-302 were evaluated every 2 cycles with the Standardized Response Criteria of the National Cancer Institute International Working Group (IWG) [24]. Efficacy evaluation for CLL was on account of IWCLL criteria [25] while for NHL, it was analyzed by IWG response criteria [26]. Patients with stable disease or partial remission were allowed to continue therapy until progressive disease or toxicity was observed.

### Data collection and assessments

Routine clinical and laboratory baseline assessments included obtaining correlative history and details of previous cancer treatment. Safety assessments included physical examination, hematology, and clinical chemistry testing (including fasting glucose and HbA concentrations), electrocardiogram and so on, during subsequent cycles. All Adverse events (AE) were graded using the Common Toxicity Criteria for Adverse Events (CTCAE) version 3.0.

Detailed pharmacokinetic were analyzed by taking blood samples before (within 1 hour) and after (every 2 hours) dosing on days 1, 28 of cycle 1. Plasma concentrations of XC-302 were measured with a validated liquid chromatography assay. Pharmacokinetic measures, such as the maximum plasma concentration (C_max_) and area under the time-concentration curve (AUC), were estimated in every patient.

### Statistical analysis

The MTD was defined as the lowest dose at which less than 1 of 6 patients experienced a DLT at that dose. Safety was assessed in all patients who received any amount of XC-302, and overall response rate, PFS were calculated in patients with at least one efficacy evaluation. Only responding patients were assessed for TTR and DOR. In calculating time-to-event variables, log-rank test and Kaplan-Meier methods were used.

## CONCLUSION

Our data support the conclusions that XC-302 in a 28-day cycle showed a tolerable safety profile. The MTD was determined to be 50 mg twice daily. Although the response rate of the drug is not as high as that of the PI3Ks inhibitors, XC-302 might be a promising therapy in patients with gastric mucosa-associated lymphoid tissue lymphoma.
